# Regulation of the Alternative Neural Transcriptome by ELAV/Hu RNA Binding Proteins

**DOI:** 10.3389/fgene.2022.848626

**Published:** 2022-02-23

**Authors:** Lu Wei, Eric C. Lai

**Affiliations:** ^1^ Key Laboratory of RNA Biology, Institute of Biophysics, Chinese Academy of Sciences, Beijing, China; ^2^ Developmental Biology Program, Sloan Kettering Institute, New York, NY, United States

**Keywords:** RNA binding protein (RBP), alternative splicing (AS), alternative polyadenylation (APA), nervous system, neuron, ELAV proteins, Hu proteins

## Abstract

The process of alternative polyadenylation (APA) generates multiple 3' UTR isoforms for a given locus, which can alter regulatory capacity and on occasion change coding potential. APA was initially characterized for a few genes, but in the past decade, has been found to be the rule for metazoan genes. While numerous differences in APA profiles have been catalogued across genetic conditions, perturbations, and diseases, our knowledge of APA mechanisms and biology is far from complete. In this review, we highlight recent findings regarding the role of the conserved ELAV/Hu family of RNA binding proteins (RBPs) in generating the broad landscape of lengthened 3' UTRs that is characteristic of neurons. We relate this to their established roles in alternative splicing, and summarize ongoing directions that will further elucidate the molecular strategies for neural APA, the *in vivo* functions of ELAV/Hu RBPs, and the phenotypic consequences of these regulatory paradigms in neurons.

## Introduction

The 3' untranslated region (3' UTR) is a hub of post-transcriptional regulation by RNA binding proteins and miRNAs, which collectively mediate diverse functional impacts including alteration of mRNA stability, directing transcript subcellular localization, modulating translational efficiency and/or changing protein function (Tian and Manley, 2017; Gruber and Zavolan, 2019). Since alternative polyadenylation (APA) causes most genes to express multiple 3' UTR isoforms, many post-transcriptional regulatory programs can be conditional. While some genes express alternative 3' UTR isoforms in constant proportion across different settings or conditions, there are numerous cases of largescale remodeling of 3' UTR usage in particular development stages, tissue- or cell-types, or in response to various genetic, environmental, or life history perturbations (Sandberg et al., 2008; Derti et al., 2012; Smibert et al., 2012; Miura et al., 2013; Xia et al., 2014; Agarwal et al., 2021). Such APA programs imply the existence of underlying molecular mechanisms that can coordinately adjust the biogenesis and/or accumulation of 3' UTR isoforms of substantial cohorts of genes, and that 3' UTR alteration should have overt functional consequences for gene regulation and function. However, to date, we know much more about the descriptive aspects of APA implementation than we do their mechanistic strategies or biological utilities.

Another dominant source of transcriptome diversity arises from alternative splicing. Compared to APA, we know more about the molecular control of alternative splice isoforms and their biological impacts (Ule and Blencowe, 2019; Bonnal et al., 2020). This is partly due to the substantial head start the splicing field has had on the APA field, and also probably due to the fact that alternative splicing often changes coding regions. In many cases, investigations of protein isoforms have a tangible hypothesis regarding differential activities, whereas it can be more challenging to decipher the physiological regulatory impacts of APA isoforms. It should be noted that certain types of APA isoforms are also associated with altered coding regions. In any case, we know of many instances in which condition-specific expression of a splicing regulator can induce coordinate and broad alterations to the transcriptome, and numerous settings where specific target splice isoforms mediate critical biology (Ule and Blencowe, 2019; Bonnal et al., 2020).

Recently, members of the conserved ELAV/Hu RNA binding protein (RBP) family have received growing attention as global mediators of both neural-specific splicing and APA programs (Carrasco et al., 2020; Wei et al., 2020; Lee et al., 2021). In this review, we will focus on newly-described global impacts of ELAV/Hu RBPs on 3' UTR landscapes, while expanding on their previously recognized roles as splicing regulators. We also summarize some of the ongoing areas of study that are needed to better understand the breadth of their impacts on the alternative transcriptome, mechanisms of action, and biological imperatives.

## The Cleavage and Polyadenylation Machinery and Connections to Splicing

**FIGURE 1:**
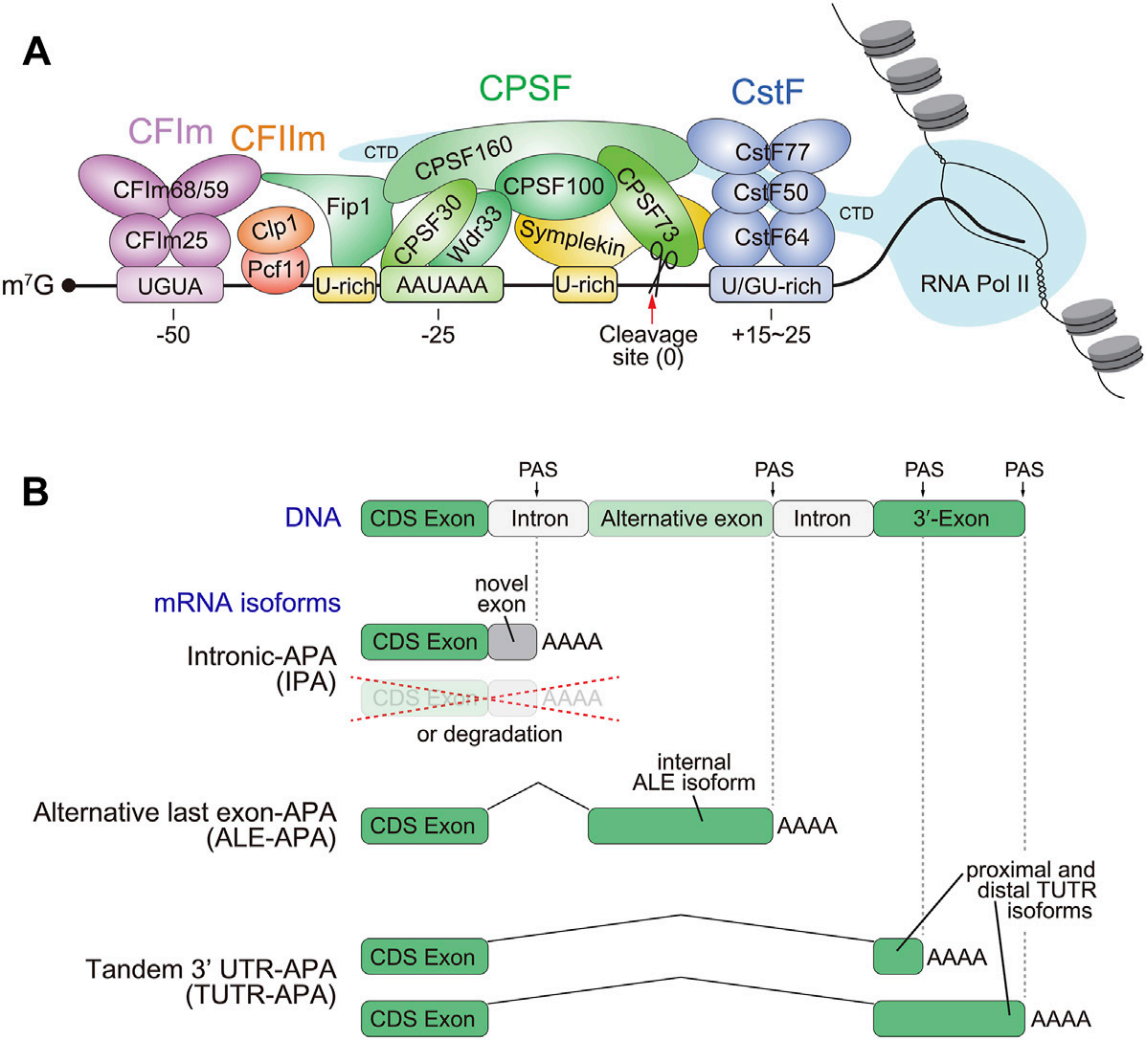
The core cleavage and polyadenylation (CPA) machinery and varieties of alternative polyadenylation (APA) isoforms. **(A)** The CPA machinery, and specifically the CPSF73 endonuclease subunit of the CPSF complex, cleaves the 3' ends of pre-mRNA transcripts, creating a site for untemplated polyadenylation that is typical of mature mRNAs. This reaction is guided by the polyadenylation signal (PAS), namely AAUAAA and related sequences. Since the PAS contains limited information, specificity can be enhanced by other local sequence motifs, which are recognized by other CPA complexes as depicted (CFIm, CFIIm and CstF). **(B)** Most pre-mRNA transcripts contain multiple functional PAS, whose alternative usage to yield distinct 3' isoforms is referred to as APA. A majority of metazoan transcripts generate more than one 3' UTR isoform, referred to as tandem 3' UTR (TUTR) isoforms. These encode the same protein but have different 3' UTR extents. Functional PAS can also be located upstream of the terminal exon in the genome. When their usage yields stable alternative transcripts, these generate alternative last exon (ALE) variants that encode different coding capacity and non-overlapping 3' UTRs. Other varieties of upstream APA, including within annotated intronic regions, can generate truncated proteins or cause the isoforms to be degraded. These are often referred to as intronic polyadenylation (IPA), although their functional utilization necessarily creates new 3' exons.

With the exception of histone mRNAs and some unique noncoding RNAs (such as *MALAT1* and *MEN β*) (Wilusz and Spector, 2010), maturation of the 3'-ends of most mRNAs relies on cleavage and polyadenylation (CPA). This involves distinct but closely-linked processes that occur co-transcriptionally: a site-directed endonucleolytic cleavage of the nascent transcript, followed by the addition of a poly(A) tail (Proudfoot, 2011). More than 50 factors participate in this two-step process (Gruber and Zavolan, 2019; Tian and Manley, 2017). In mammals, the CPA machinery can be further divided into four functional subcomplexes (Figure 1A). 1) The Cleavage and Polyadenylation Specificity Factor (CPSF) complex consisting of six major subunits CPSF160, CPSF100, CPSF73, CPSF30, WDR33 and Fip1, is responsible for recognition of poly(A) signals (PAS) and cleavage of pre-mRNA (Casanal et al., 2017; Clerici et al., 2017; Clerici et al., 2018; Sun et al., 2018). In particular, CPSF30 and WDR33 directly bind AAUAAA motifs (Chan et al., 2014; Schonemann et al., 2014), which guides the endonuclease CPSF73 to cleave the primary transcript ∼10–30 nt downstream (Ryan et al., 2004; Mandel et al., 2006), generating the site of untemplated polyadenylation. 2) The Cleavage Factor complexes, CFIm and CFIIm. Of these, CFIm is better understood, and is composed of CFIm25 (also known as CPSF5 or Nudt21) and CFIm68 (also known as CPSF6). Both factors form homodimers, with CFIm25 recognizing the UGUA upstream element to promote 3' end formation (Yang et al., 2011). Multiple UGUA elements can further stimulate 3' cleavage, and the paralog CFIm59 can substitute for CFIm68 function in the complex (Zhu et al., 2018). CFIIm is composed of the Pcf11 and Clp1, and this complex may help bridge CFIm and CPSF (de Vries et al., 2000), and interact with the RNA Pol II C-terminal domain (Barilla et al., 2001; Licatalosi et al., 2002). However, the precise role of CFIIm in the CPA machinery requires further study (Schafer et al., 2018). 3) The Cleavage stimulation Factor (CSTF) complex, including Cstf50 (CSTF1), Cstf64 (CSTF2) and its paralog Cstf64τ, and Cstf77 (CSTF3), binds a U/GU-rich region downstream of a cleavage site and enhances CPA (Bai et al., 2007; Luo et al., 2013; Yao et al., 2013; Grozdanov et al., 2018; Yang et al., 2018). 4) Additional factors such as poly(A) polymerase, poly(A) binding proteins, function during synthesis and protection of poly(A) tails during CPA, while scaffold and auxiliary factors like Symplekin and RBBP6 (Sullivan et al., 2009; Xiang et al., 2010; Di Giammartino et al., 2014; Tatomer et al., 2014), interact with RNA polymerase II and help connect CPA with other types of co-transcriptional and post-transcriptional gene regulation processes of nascent transcripts.

More generally, as 3'-end formation is a co-transcriptional process, one can imagine that other chromatin or transcriptional mechanisms could influence 3' end formation, either through indirect impacts or via specific functional couplings. For example, epigenetic modifications and nucleosomal organization (Huang et al., 2013; Soles and Shi, 2021), Pol II elongation dynamics (Fong et al., 2015; Yang et al., 2016; Geisberg et al., 2020), even RNA export factors (Johnson et al., 2011; Tran et al., 2014) have all been linked to efficient and/or alternative 3' end formation. Of note, there are particularly rich couplings between splicing factors with 3' end maturation (Millevoi et al., 2002; Albulescu et al., 2012; Mora Gallardo et al., 2021; Reimer et al., 2021; Schwich et al., 2021). Reciprocally, some CPA factors have also been shown to influence splicing (Misra et al., 2015). The relationship of these processes is further emphasized by the domain similarity of CFIm68 with spliceosomal SR proteins (Ruegsegger et al., 1998), and their analogous mechanisms (Zhu et al., 2018).

The extent to which coupling of splicing and CPA is direct is a topic of ongoing studies; however, mechanistic insights are emerging. For example, splicing factors SRSF7 promotes proximal PAS usage, accumulates at such sites, and uses its RS domain to interact with CPA factor Fip1. On the other hand, splicing factor SRSF3 promotes distal PAS usage by maintaining productive splicing of CFIm68 (CPSF6) (Schwich et al., 2021). Since both splicing and CPA regimes are highly regulated and subject to alternative outcomes, such precedents suggest that these processes might be coordinated to generate specific combinations of sequences in mature mRNAs. Coordinated splicing across multiple exons has been observed (Tilgner et al., 2018; Drexler et al., 2020), although in general it is not clear how this is accomplished mechanistically. Moreover, an alternative internal exon splicing choice is coupled to an alternative 3' UTR choice at *Drosophila Dscam1*, such that distinct protein-encoding isoforms intrinsically bear different post-transcriptional content (Zhang et al., 2019). It is likely that further applications of long-read sequencing technologies (Gupta et al., 2018; Legnini et al., 2019) will reveal additional examples of coordinated alternative splicing and CPA, whose existence implies further mechanistic complexities in mRNA processing that remain to be elucidated.

## Locations of Poly(A) Signals and Classes of APA Isoforms

CPA is initiated by recognizing poly(A) signals (PAS) on nascent transcripts. PAS are located 10–30 nt upstream of cleavage sites, and defined by the canonical hexamer AAUAAA (Proudfoot and Brownlee, 1976), along with a number of suboptimal but functional variants (e.g., AUUAAA and AAUAUA, etc.) (Hu et al., 2005; MacDonald and Redondo, 2002; Sheets et al., 1990). Since simple sequence matches to PAS are prevalent across primary transcription units, including not only within 3' UTRs but also within coding exons, 5' UTRs and introns, there are many potential opportunities for CPA to occur at more than one site, resulting alternative cleavage and polyadenylation (APA) (Figure 1B). Genomewide profiling indicates that ∼75% of human genes have two 3' isoforms, while about half of all genes utilize three or more PAS (Tian and Manley, 2017; Gruber and Zavolan, 2019). Even with extensive 3'-end catalogues to date, these probably still underestimate true breadth of functional PAS in transcriptomes. For example, some genes may produce unannotated isoforms that are highly cell- or condition-specific, and the role of some PAS is to eliminate certain isoforms which may consequently not accumulate in normal settings. Thus, additional profiling, including from genetic conditions that protect from RNA degradation, will continue to reveal new transcript isoforms.

APA within a given 3' UTR, also referred to as tandem 3' UTR-APA (TUTR-APA), generates mRNA isoforms with the same coding region but with different 3' UTR lengths (Figure 1B). The alternative 3' UTRs often harbor binding sites for various regulatory factors, i.e., RNA binding proteins (RBPs), microRNAs (miRNAs), long non-coding RNAs (lncRNAs), etc. Collectively, these influence nuclear export, stability and/or localization of individual mRNA isoforms, and further impact overall gene activity via translation efficiency, protein localization and activity, and so forth (Wu and Bartel, 2017; Vejnar et al., 2019). For example, the different mRNA isoforms of brain-derived neurotrophic factor (BDNF) gene generated by TUTR-APA, exhibit distinct localization in neurons: mRNA isoform bearing long 3' UTR preferentially localizes in dendrites, while the short 3' UTR isoform remains in cell body (An et al., 2008). Such localization differences facilitate local BDNF protein synthesis for particular dendrite-specific protein functions (Lau et al., 2010; Liao et al., 2012). A further indication of the general importance of neural APA is the fact that heterozygosity for CPA factor *CFIm25* (*Nudt21*) alters 3' isoforms in the hippocampus and yields behavioral defects (Alcott et al., 2020). The dose sensitivity of CFIm25 suggests that mild changes in CPA efficiency might be sufficient to cause intellectual disability or behavioral phenotypes in humans.

On the other hand, the use of more upstream PAS, in either exons or introns, will result in mRNA isoforms with distinct exonic content (Figure 1B). The functional consequences of this are varied, depending on whether the APA isoforms are stable or unstable. The former are classifiable as alternative last exon (ALE) isoforms, whereas usage of internal pA sites that do not yield stable mRNA (perhaps due to isoform destabilization) would appear as intronic polyadenylation (IPA) (Lee et al., 2018; Singh et al., 2018). Stable ALE isoforms may encode distinct C-termini that confer corresponding distinct protein functions (Taliaferro et al., 2016; Tien et al., 2017), or may yield truncated dominant negative isoforms to inhibit full-length protein activities (Mueller et al., 2016). The nervous system is rich in genes that express multiple ALE isoforms (Koushika et al., 1996; Lisbin et al., 2001; Lee et al., 2021), which contain different coding information as well as non-overlapping 3' UTRs that may confer distinct regulatory paradigms. A particularly compelling example is the *Drosophila* gene *lola*, which plays diverse roles in neural development and differentiation (Goeke et al., 2003; Southall et al., 2014; Sato et al., 2019), as well as in certain other tissues. *lola* undergoes a high degree of alternative processing to generate >80 mRNA isoforms, of which 20 are non-overlapping ALE isoforms with distinct 3' UTRs; 17 of which encode different C-terminal zinc fingers. Systematic deletions of individual *lola* ALEs demonstrates that several are required for various aspects of neural differentiation or maintenance (Dinges et al., 2017).

The usage of upstream PAS can also generate nonsense isoforms that are cleared by the Nonsense Mediated Decay (NMD) machinery. For example, core CPA genes *pcf11* and *cstf3* genes are both subject to intronic PAS to generate truncated isoforms that reduce effective CPA activity (Luo et al., 2013; Kamieniarz-Gdula et al., 2019; Wang R. et al., 2019). Thus, generation of an appropriate landscape of 3' ends requires upstream/intronic APA of core APA factors, a classic autoregulatory loop.

## The Unique Extended 3' Untranslated Region Landscape of Neural Tissues and Neurons

APA is widespread across diverse multicellular eukaryotes, including both animals and plants (Tian and Manley, 2017; Gruber and Zavolan, 2019). The expression of 3' isoforms is often differentially regulated across development, amongst different tissues and cell types, and/or in response to environmental or metabolic changes. This implies that alternative post-transcriptional regulatory programs are tailored for cohorts of genes, and that there should be underlying mechanisms that are shared across regulated loci.

A particularly notable setting is the expression of hundreds of extended 3' UTR isoforms in neural settings (Miura et al., 2014), both in vertebrates and invertebrates (Hilgers et al., 2011; Shepard et al., 2011; Smibert et al., 2012; Ulitsky et al., 2012; Lianoglou et al., 2013; Miura et al., 2013). Most of the evidence comes from RNA-seq or 3'-seq profiling from tissues rich in neurons (e.g. dissected CNS tissue), showing their expression of long 3' isoforms that are absent from other tissue or cell types. We may infer that CNS-specificity reflects long neural isoforms, but most of the direct evidence for these comes from analyzing the directed differentiation of pluripotent cells into neurons (Shepard et al., 2011; Lee et al., 2021). Rapid advances in single-cell RNA sequencing (scRNA-seq) hold great promise to decipher cell-specific isoforms. Indeed, the first scAPA analyses have recently been reported (Shulman and Elkon, 2019; Gao et al., 2021; Li et al., 2021), and document 3' UTR lengthening in identified neuron subtypes (Agarwal et al., 2021; Yang et al., 2021).

Both the breadth and magnitude of extended 3' UTR neural isoforms are remarkable, with hundreds of genes exhibiting longer 3' UTRs in nervous system compared to other settings, with lengths up to ∼20 kb 3' UTRs for certain genes in both *Drosophila* and mammals (Hilgers et al., 2011; Smibert et al., 2012; Miura et al., 2013; Sanfilippo et al., 2017a). These initial annotations of neural extended 3' UTRs converted many megabases of formerly “intergenic” space into each of the transcriptomes of flies, mice and humans, and they contain numerous miRNA and RBP sites. These have predominantly been annotated through a combination of short read RNA-seq and directed 3'-seq protocols, neither of which reports directly on full-length transcripts. In particular, the existence of such extremely long, continuous exons is not typically permitted by many *de novo* transcriptome assemblers, and requires different methodology (Shenker et al., 2015) along with systematic visual inspection of gene models from the primary mapped data (Miura et al., 2014). Thus, to date, Northern blotting has been the most definitive method for confirming the tissue-specific accumulation of the longest neural isoforms (Smibert et al., 2012; Miura et al., 2013), which have never been captured in cDNA libraries. Strategies for long read sequencing are improving (Gupta et al., 2018; Legnini et al., 2019), but the isolation of full-length long transcripts is limited by reverse transcription. Direct RNA sequencing using nanopores is another appealing alternative (Wang et al., 2021), and as throughput increases, this may eventually be critical to largescale discovery and interrogation of transcript isoforms with complex primary processing (splicing and/or APA).

## The ELAV/Hu Family of Metazoan RNA Binding Proteins: Functions and Evolution

How is the distinctive 3' UTR landscape of neurons determined? In general, mechanisms of APA in various settings include dynamic kinetics of RNA polymerase II, modulation of CPA factors, or trans-acting factors that might alter CPA activity (Miura et al., 2014). In principle, several of these strategies could operate in the same cell. However, the neural-specific expression of many factors, including of RBPs, may suggest that trans-acting neuronal factors may help drive the extended 3' UTR landscape in this unique cell type. For example, mammalian NOVA1 and NOVA2 are neural-enriched RNA-binding proteins that control mRNA alternative splicing (Licatalosi et al., 2008), but NOVA2 also regulates APA by binding nearby proximal PAS in developing mouse brain (Ule et al., 2006). Another early-recognized candidate involves members of the ELAV/Hu RBP family, which are conserved across animals (Bronicki and Jasmin, 2013; Colombrita et al., 2013). This family comprises multifunctional RNA binding proteins, most of which are neural-restricted, and have been connected to diverse regulatory paradigms including alternative splicing, alternative 3' UTRs, mRNA stability, translational efficiency, and localization.

**FIGURE 2:**
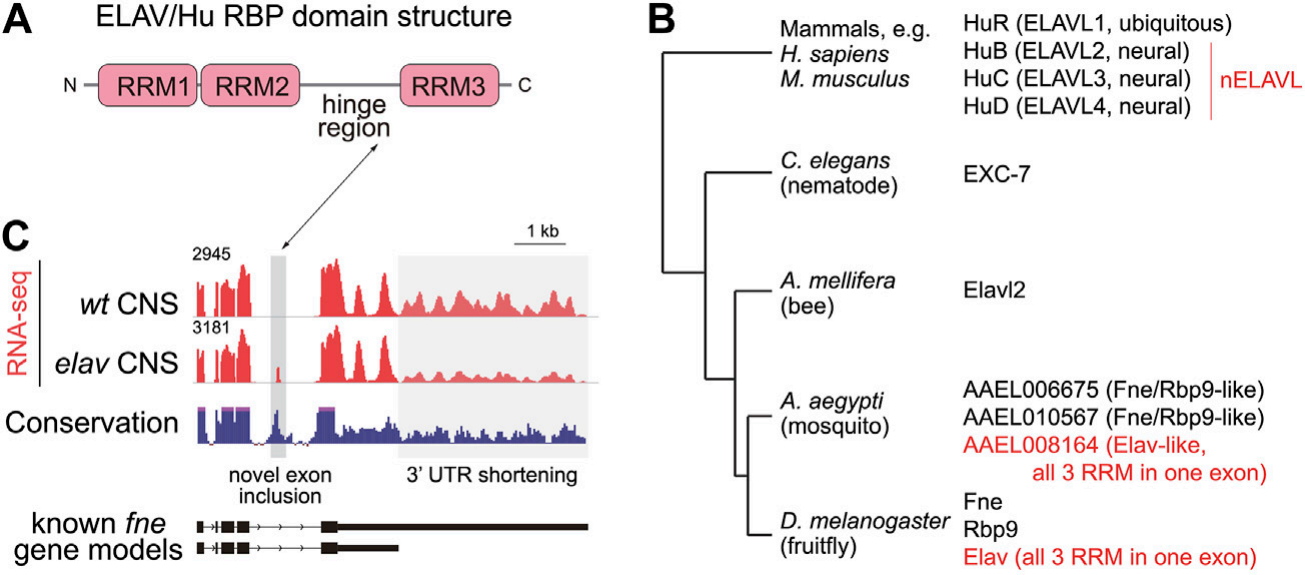
Phylogeny and alternative processing of ELAV/Hu RNA binding proteins (RBPs). **(A)** All members of the ELAV/Hu family share a common structure including 3 RNA recognition motifs (RRMs) and a hinge between RRM2 and RRM3. **(B)** Distinct numbers of ELAV/Hu RBP genes in different metazoan species suggest that multiple amplifications of the family occurred during evolution. Note that while many homologs are referred to as “Elav-like,” based on the founding member from flies, *Drosophila* Elav is actually an evolutionarily derived retrogene. Phylogenetic tree is modified from (Samson, 2008). **(C)** Cross-regulation of *Drosophila fne* mRNA isoforms by Elav. RNA-seq data (red tracks) from wildtype CNS shows substantial expression of an extended 3' UTR isoform of *fne*, while *elav* mutant CNS expresses a shorter *fne* 3' UTR isoform that also includes a novel microexon that can direct Fne towards the nucleus.

The founding member of this family came from *Drosophila*, where *elav* (*embryonic lethal abnormal vision*) was initially studied genetically as a locus that is required for viability as well as the differentiation and survival of certain neural tissues, including the eye (Campos et al., 1985; Homyk et al., 1985). Cloning of *elav* revealed a factor encoding 3 RNA recognition motifs (RRMs), with a hinge region connecting RRM2 and 3 (Campos et al., 1987; Bier et al., 1988; Robinow et al., 1988) (Figure 2A). *Drosophila* encodes several proteins with a similar overall domain arrangement (Figure 2B), including two clear paralogs of Elav, namely Rbp9 (RNA binding protein 9) (Kim and Baker, 1993) and Fne (Found in neurons) (Samson and Chalvet, 2003). All of the fly proteins are strongly upregulated in neurons and/or exhibit neural-related defects, although Rbp9 is also female sterile (Kim-Ha et al., 1999). Knowledge of these fly factors provided context for the finding that human homologs of Elav proved to be human auto-antigens in paraneoplastic encephalomyelitis, a rare class of neurological syndrome (Graus et al., 1986; Szabo et al., 1991; King et al., 1994; Sakai et al., 1994; Ma et al., 1996). These are often associated with small cell lung carcinomas, in which ectopic Hu proteins in cancer cells are recognized by auto-antibodies; Hu refers to the first initials of an affected patient. These go on to attack neurons, which are the predominant locations of expression of three of the four mammalian ELAV/Hu proteins.

*Drosophila* Elav appears to mark most if not all neurons across development (Robinow et al., 1988; Robinow and White, 1991), and owing to the existence of highly-specific monoclonal antibodies, is the most broadly used neuronal marker in this species. Transgenic and clonal studies reveal that *elav* loss results in severe defects in cell composition and function of the eye (Campos et al., 1985; Homyk et al., 1985; Koushika et al., 1996), while null and temperature-sensitive mutants showed additional requirements in synapse formation, axon guidance and neural activity (Simionato et al., 2007; Haussmann et al., 2008). Curiously, amongst the three fly paralogs, Elav is the only Hu family protein that is individually required for viability. Fne and RBP9 mutants are viable, but exhibit specific defects. Loss of *fne* is compatible with viability (Zaharieva et al., 2015), but yields defects in the morphology of some CNS structures, the differentiation of certain PNS neurons (Alizzi et al., 2020), and certain aberrant behaviors (Zanini et al., 2012). Mutants of *rbp9* are similarly viable but exhibit blood-brain barrier defects and shortened lifespan (Kim et al., 2010; Toba et al., 2010), in addition to exhibiting female sterility and oogenesis defects (Kim-Ha et al., 1999). Because Elav is predominantly nuclear while Fne and Rbp9 are mostly cytoplasmic, the markedly different viability and neural phenotypes of *elav* compared to *fne* or *rbp9* mutants were long taken to reflect that the three ELAV/Hu RBPs play distinct roles in *Drosophila*. On the other hand, rigorous *in vivo* genetic analyses indicate that ectopic Fne and Rbp9 can at least partially rescue *elav* null settings (Zaharieva et al., 2015).

Vertebrates encode 4 ELAV/Hu family RNA binding proteins, named Elavl1 (HuR), and Elavl2/3/4 (HuB/C/D) (Figure 2B). HuR is ubiquitously expressed across different tissues, while HuB/C/D are largely restricted to the nervous system, and sometimes referred as nELAVL proteins (King et al., 1994; Akamatsu et al., 1999). Mouse *HuR* knockouts are embryonic lethal (Ghosh et al., 2009; Katsanou et al., 2009), and conditional analyses reveal roles across a wide range of biological settings, including but certainly not limited to angiogenesis (Chang et al., 2014), germinal centre response (Osma-Garcia et al., 2021), adipogenesis (Siang et al., 2020), and colon cancer (Akaike et al., 2014). In contrast, mutants of neural ELAV/Hu paralogs such as HuC (Ince-Dunn et al., 2012) and HuD (Akamatsu et al., 2005) are viable. Their more subtle defects correlate with the restricted neural expression domains of nELAVL factors. Nevertheless, neural Hu RBPs are clearly required, since both of these individual knockouts exhibit specific behavioral defects, and the *HuC/D* dKO mice die shortly after birth (Ince-Dunn et al., 2012). Overall, nELAVL RBPs are implicated in promoting neuronal identity and maturation (Akamatsu et al., 1999; Grassi et al., 2018), maintaining neural activities (Ince-Dunn et al., 2012), and shaping neural-specific transcriptome features (Scheckel et al., 2016) including stabilization of neural targets (Zybura-Broda et al., 2018; Lu et al., 2021). Beyond normal development, dysfunction of nELAVL RBPs has now been linked to various neurological diseases (Berto et al., 2016; Bowles et al., 2021; Diaz-Garcia et al., 2021).

There are additional important, but not well-appreciated, features of the ELAV/Hu phylogeny, even though they were described some time ago (Samson, 2008). First, the existence of multiple ELAV/Hu paralogs in both *Drosophila* and mammals might suggest there could be orthology of specific fly and mammalian family members (Figure 2B). However, this is likely not the case. In particular, while multiple dipteran and at least some lepidopteran species (i.e., in the superorder *Panorpida*), other arthropods, as well as *C. elegans*, encode only a single member (Samson, 2008). Thus, the genomic amplification of ELAV/Hu members likely occurred several times during metazoan evolution. Second, although many homologs in other species are named in the literature as Elav-like genes, the founding member Elav is clearly an evolutionarily derived copy. This is evident from the fact that the coding region of *Drosophila elav* is contained in a single exon, presumably resulting from retrogene insertion, and actually resides in an intron of the *arginase* gene. Most Dipterans harbor an analogous family member lacking introns within their coding regions, although not in the syntenic position (i.e., not within *arginase*); it is conceivable these are orthologs (Samson, 2008). For this reason, we prefer using the term “ELAV/Hu family,” since vertebrates do not have technically harbor an Elav ortholog. Other *Drosophila* ELAV/Hu genes (*fne* and *rbp9*) harbor introns, and some of these reside in similar positions as the vertebrate ELAV/Hu genes (Samson, 2008). However, none of these match the vertebrate intron/exon positions as well as with other non-Dipteran arthropod homologs. This again supports the concept that ELAV/Hu genes diversified recently within the lineage leading to *Drosophila* species.

Recently, it was clarified that *Drosophila fne* gene encodes both cytoplasmic and nuclear isoforms, generated by alternative splicing, and that a newly-recognized *fne* microexon is preserved in other arthropods (Carrasco et al., 2020; Wei et al., 2020) (Figure 2C). This may suggest that although *Drosophila fne* knockouts exhibit only mild defects on their own, Fne actually harbors features of an ancestral ELAV/Hu member. Consistent with this, very recent studies of the single honeybee member ELAVL2 (which shares the *Drosophila Fne* microexon), reveals highly complex alternative splicing and variable levels of nuclear and cytoplasmic proteins in different settings (Ustaoglu et al., 2021). This is consistent with the notion that a single ancestral ELAV/Hu locus might combine functions that are separated into multiple family members in other species. We can infer that the ELAV/Hu member in the protostome ancestor had important neural roles, since *C. elegans* homolog EXC-7 is involved in synaptic transmission (Loria et al., 2003), and an Aplysia ELAV member mediates long term memory formation (Mirisis et al., 2021).

Studies using *in vitro* binding assays and *in vivo* high-throughput profiling reveal that ELAV/Hu proteins can bind similar U/AU-rich regions enriched in introns and 3' UTRs to regulate pre-mRNA processing (Peng et al., 1998; Ray et al., 2013; Zaharieva et al., 2015; Scheckel et al., 2016). We now turn attention to recent discoveries on the roles of ELAV/Hu family RBPs in regulating neural-specific transcriptome features in both *Drosophila* and mammals.

## ELAV/Hu Family RBPs Regulate Alternative mRNA Splicing

Amongst different ELAV/Hu family members, mammalian HuR is perhaps the most well-studied member, with diverse roles in co-transcriptional and post-transcriptional gene regulation of mRNAs. HuR protein can shuttle from nucleus to the cytoplasm (Fan and Steitz, 1998a; Fan and Steitz, 1998b), whose functions are regulated by dynamic subcellular localization (Wang Y. et al., 2019), which presumably determines its capacity to regulate mRNA processing vs. control cytoplasmic mRNA stability (Lebedeva et al., 2011; Mukherjee et al., 2011). Under normal physiological conditions, HuR is predominantly localized in nucleus to primarily mediate alternative mRNA splicing (Izquierdo, 2008; Wang et al., 2010). For example, multiple ELAV/Hu factors can potentially cross-regulate promote exon inclusion in the *HuD* paralog (Wang et al., 2010), while HuR promotes exon skipping in the apoptosis receptor *Fas* (Izquierdo, 2008). When cells experience stress, HuR can translocate to the cytoplasm where it stabilizes and/or promotes the translation of target mRNAs (Peng et al., 1998; Mazan-Mamczarz et al., 2003). Early on, HuD was also noticed to be both nuclear and cytoplasmic, and implicated as a post-transcriptional positive regulator of *N-myc* (Lazarova et al., 1999).

**FIGURE 3:**
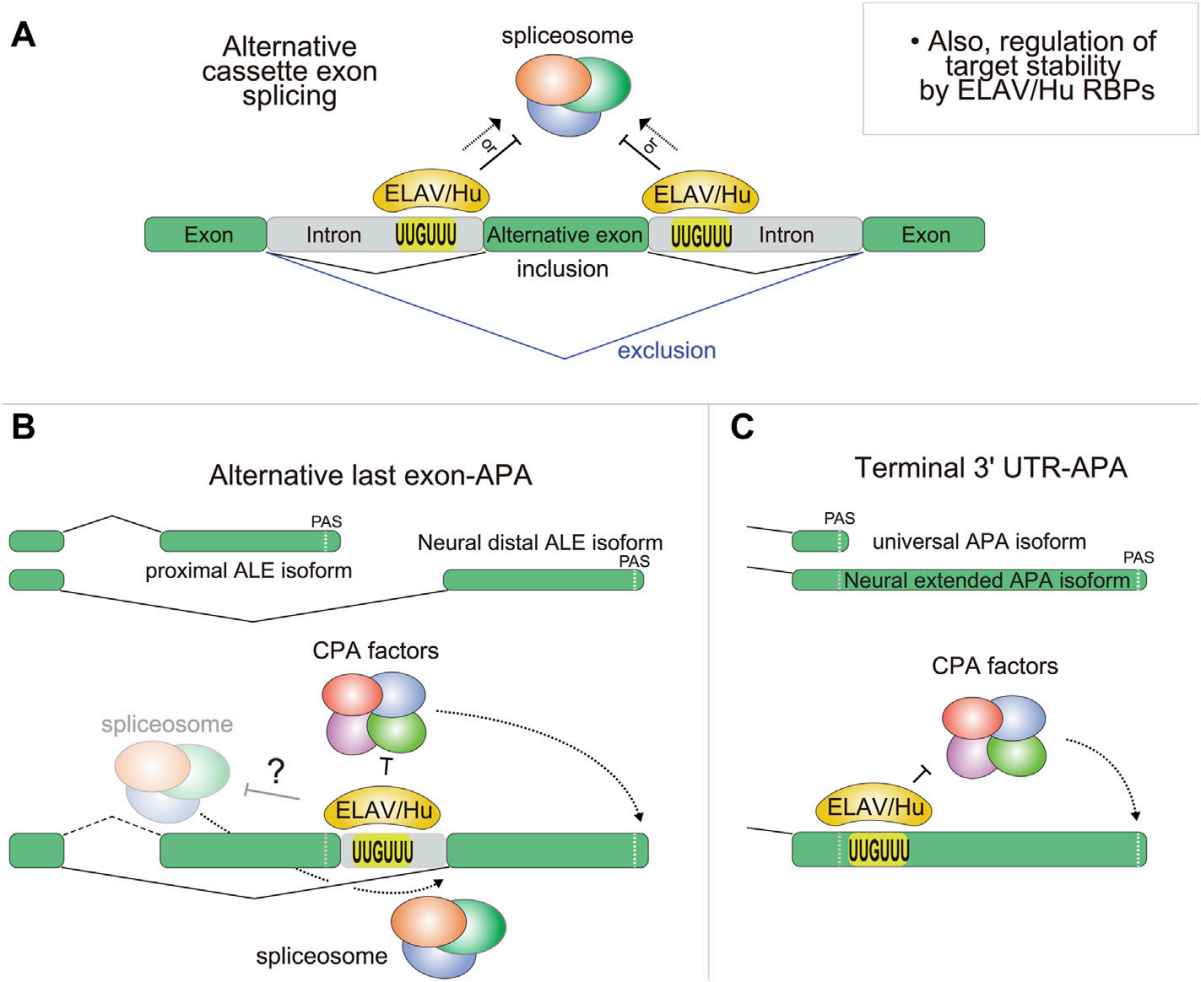
Regulation of alternative splicing and 3' UTRs by ELAV/Hu RBPs. This figure summarizes effects on mRNA isoform generation, but ELAV/Hu factors also affect target mRNA levels. **(A)** The genetic manipulation of both fly and mammalian ELAV/Hu RBPs causes largescale changes in the expression of alternative internal splicing isoforms, causing some cassette exons to be included while others are excluded. Presumably, the local binding of ELAV/Hu RBPs flanking the regulated exons impacts the spliceosome machinery, but the details that control different functional outcomes remain to be understood. It is also possible that some changes in alternative splicing observed upon manipulation of ELAV/Hu factors are indirect effects. **(B)** *Drosophila* ELAV/Hu RBPs globally shift the usage of proximal alternative last exon (ALE) isoforms towards distal ALE isoforms in neurons. While this can be viewed as splicing regulation, available evidence suggests that ELAV/Hu RBPs control this by suppressing PAS usage at proximal ALE 3' termini, permitting transcription and splicing into distal ALE exons. It remains to be seen if this regulation involves direct effects on the spliceosome (designated as “?”). **(C)** In *Drosophila*, multiple ELAV/Hu members drive both global 3' UTR lengthening by preferentially binding downstream of proximal PAS, presumably to inhibit cleavage and polyadenylation (CPA) activity at proximal PAS. However, the contribution of post-transcriptional mechanisms (e.g., differential control of mRNA isoform stability by ELAV/Hu RBPs) is not yet ruled out.

As ELAV/Hu studies have moved into the genomic age, it became *de rigueur* to use RNA-seq for comprehensive documentation of effects on alternative splicing (Figure 3A). Indeed, broad splicing changes are observed upon manipulation of ELAV/Hu factors, including with HuR (Akaike et al., 2014; Chang et al., 2014; Osma-Garcia et al., 2021; Sena et al., 2021) and neural ELAV members (Ince-Dunn et al., 2012; Berto et al., 2016; Scheckel et al., 2016; Bowles et al., 2021). At least a portion of these may be direct targets, as supported by corresponding evidence of ELAV/Hu occupancy on flanking intron regions (Lebedeva et al., 2011; Mukherjee et al., 2011; Ince-Dunn et al., 2012; Scheckel et al., 2016). However, it can be difficult to distinguish direct from indirect effects in steady-state genomic data. A recent study addressed this challenge by dynamically profiling the response to immune stimulation (Rothamel et al., 2021). These data revealed a redistribution of HuR from introns to 3' UTRs during this process, accompanied by stabilization of its targets (particularly amongst interferon-stimulated genes). Thus, care should be taken in interpreting the functional basis of genes with altered splicing or abundance in mutants of ELAV/Hu factors.

In *Drosophila*, research on Elav in post-transcriptional gene regulation began 25 years ago, when the genes *erect wing (ewg)*, *neuroglian* (*nrg*), and *armadillo* (*arm*) were recognized to exhibit unique neural-specific alternative pre-mRNA splicing that was regulated by Elav (Koushika et al., 1996; Koushika et al., 2000; Lisbin et al., 2001; Soller and White, 2003). Neural regulation of *arm* occurs via exclusion of an internal cassette exon, but splicing regulation of *ewg* and *nrg* is of the less common alternative last exon (ALE) variety (Figure 3B). Non-neural isoforms of these genes utilize an upstream PAS, whereas these sites are bypassed in neural isoforms, permitting splicing to a downstream ALE in neurons. Thus, as mentioned in the introduction, ALE splicing can be a form of alternative PAS usage. Mechanistic studies confirmed that *ewg* and *nrg* were directly regulated by Elav binding to U-rich regions in introns to competitively inhibits 3'-end processing at proximal ALE termini (Haussmann et al., 2011; Lisbin et al., 2001; Soller and White, 2003; 2005).

One may reasonably speculate whether the Elav paralogs Rbp9 and Fne possess similar regulatory activities in neurons. Experimental tests showed that ectopic expression of Rbp9 or Fne can partially compensate for conditional ablation of Elav in the developing eye (Zaharieva et al., 2015); however, single or double mutants of *rbp9* and *fne* have only mild effects on neural development and did not apparently alter the few known Elav-dependent splicing targets. This may correlate with the fact that Fne and Rbp9 are predominantly localized in cytoplasm while Elav is primarily in nucleus. For these reasons, relatively little research has been conducted on neural regulatory functions of Fne and Rbp9. However, it is also perhaps surprising that little has been done to determine transcriptome-wide defects in ELAV/Hu mutants in *Drosophila*. Recently, this was addressed by our group and the Hilgers group (Carrasco et al., 2020; Wei et al., 2020; Lee et al., 2021).

Transcriptome profiling of individual mutants or combinations thereof, showed that multiple ELAV/Hu members co-determine neural alternative splicing in *Drosophila*. In particular, while profiling of *elav* mutants showed certain shifts to both inclusion and exclusion of cassette exons, the effects were much more pronounced in *elav, fne* double mutants. There was also an apparent developmental effect, in that the effects in late stage embryos (Carrasco et al., 2020) were far less pronounced than from early larval CNS (Lee et al., 2021). About 100 total genes exhibited altered splicing in the former dataset whereas ∼800 genes were affected in the latter dataset. This might be due in part to the fact that dissected CNS contains a higher proportion of neurons than does whole embryos, which likely increases signal when inferring neural gene regulation from mixtures of cell types. Another consideration is that Elav is dominant amongst ELAV/Hu family members in embryos, but Fne levels increase substantially in early larval stages (with Rbp9 peaking later still) (Zaharieva et al., 2015; Wei et al., 2020). Thus, there may be broader aggregate effects of ELAV/Hu members on neural gene regulation as neurons mature. However, it is not simply the case that changes in mRNA processing are only due to indirect effects on neural maturation, since Elav and Fne are sufficient to induce effects on alternative splicing in a heterologous cultured cell system (S2 cells) (Lee et al., 2021). In addition, there is evidence for occupancy of Elav in the flanking introns of at least some of its splicing targets, in addition to enrichment for U-rich sequences that resemble known ELAV/Hu binding sites (Carrasco et al., 2020; Lee et al., 2021). Of note, ectopic Rbp9 also induced similar effects in transcriptome studies (Wei et al., 2020; Lee et al., 2021), suggesting that it may also be relevant to the neural alternative transcriptome in later stages.

Strikingly, *fne* is amongst the minority of genes whose splicing is strongly altered in *elav* mutants, which cause the accumulation of a novel short exon in *fne* in both embryos and in larval CNS (Carrasco et al., 2020; Wei et al., 2020). Thus, unlike the bulk of splicing targets that require the combined activities of Elav and Fne, the alternative processing of *fne* is extremely sensitive to Elav activity, not only of alternative splicing but also 3' UTR elongation (Figure 2C). This implies cross-regulation of ELAV/Hu family members, as hinted in mammalian studies (Mansfield and Keene, 2012; Wang et al., 2010). But it is more complex than this, since the alternative Fne exon is very short (encoding 15 amino acids) and was in fact unannotated, despite a long history of systematic cataloguing of *Drosophila* transcripts (Smibert et al., 2012; Brown et al., 2014; Westholm et al., 2014; Sanfilippo et al., 2017b). The Fne microexon is conserved in other insects, and resides near the hinge between RRM2 and RRM3 (Figure 2C). The hinge-adjacent region undergoes alternative splicing in some mammalian ELAV/Hu genes, and controls shuttling between nucleus and cytoplasm (Fan and Steitz, 1998a). Such regulation of *fne* is consequential, since Fne protein is substantially cytoplasmic, but the inclusion of its microexon causes it to be preferentially nuclear (Carrasco et al., 2020; Wei et al., 2020). Therefore, there is a hierarchy of nuclear ELAV/Hu RBPs in *Drosophila* that can be regulated dynamically by splicing. This may relate to the ancestral state of insect genomes, which may have only an Fne-type family member that executes both nuclear and cytoplasmic functions (Samson, 2008; Ustaoglu et al., 2021).

Finally, we note that ALE splicing is also broadly and reciprocally disrupted in loss-of-function and gain-of-function conditions of Elav and Fne (Carrasco et al., 2020; Lee et al., 2021). However, unlike internal cassette exon splicing, where relatively similar cohorts of genes preferentially undergo either inclusion or exclusion upon manipulation of ELAV/Hu RBPs, ALE splicing is directionally affected as a category. That is, in gain-of-function conditions in S2 cells, dozens of genes shift towards distal 3' ALE isoform usage, whereas in loss-of-function conditions in embryos or larval CNS, dozens of genes shift towards proximal 3' ALE isoform usage. As noted, two of the three loci that were historically known to be ELAV regulated happen to be ALE splicing targets, even though as a class these are far less abundant than are alternative cassette exons, and these genes (*nrg* and *ewg*) were found to be regulated by inhibition of 3' end processing at PAS of proximal ALE isoforms (Lisbin et al., 2001; Soller and White, 2003). Together, these observations establish ELAV/Hu RBPs as major remodelers of the neural-specific alternative transcriptome, and furthermore, their broad roles in ALE regulation suggest they could have general relevance for neural APA.

## ELAV/Hu Family RNA Binding Proteins Promote the Extended Neural 3'-End Landscape

Earlier, by careful analysis of deep stranded mRNA-seq data, we uncovered global neural TUTR-APA extension isoforms in both mammalian (mouse and human) brains and *Drosophila* CNS (Smibert et al., 2012; Miura et al., 2013; Sanfilippo et al., 2017b); an analogous scenario emerged from developmental microarray profiling during *Drosophila* embryogenesis (Hilgers et al., 2011). For nearly a decade, the underlying mechanisms that directs the distinctive and conserved neural extended 3'-end landscape remained elusive. However, several studies pointed to roles for ELAV/Hu RBPs. For example mammalian HuR and nELAVL proteins can promote neural 3' UTR extension of *HuR* mRNA, which is inherently less stable and translationally suppressed during neuronal differentiation (Dai et al., 2012; Mansfield and Keene, 2012). Subsequently, HuC was reported to regulate APA events during differentiation of inhibitory neural progenitors ( Grassi et al., 2018 ). Additional *in vitro* assays confirmed HuR and nELAVL proteins selectively block both cleavage and polyadenylation at sites containing U-rich sequences, which compromised the direct interactions between functional *cis*-elements with core CPA factors of Cstf64 and CPSF160 (Zhu et al., 2007). However, it is largely unclear how if mammalian ELAV/Hu RBPs play broader roles in neural APA.

Studies of *elav* mutant embryos provided evidence that Elav mediates neural 3' UTR lengthening (Hilgers et al., 2012). However, considering the overlapping functions of ELAV/Hu members described above, a more comprehensive appreciation of their impacts on APA could only be gained using the datasets from gain- and loss-of-function of *Drosophila* ELAV/Hu family members. In total, analysis of S2 cells, embryos and early larval CNS revealed hundreds of genes whose 3' UTR isoforms are directionally controlled by ELAV/Hu RBPs (Carrasco et al., 2020; Wei et al., 2020; Lee et al., 2021). These follow the same genetic dependencies as with neural splicing, in that *elav, fne* double mutants are far more severe APA defects than single mutants (with *elav* showing modest effects and *fne* showing lacking substantial effects). Moreover, all three family members were capable of conferring globally lengthened 3' UTRs in S2 cells. Although these cells are not converted into neurons *per se*, they do exhibit largescale shifts to neural-preferred cassette exons, distal ALE usage, and extended 3' UTRs, and often utilize the particular isoform expressed in the nervous system (Wei et al., 2020; Lee et al., 2021).

Mechanistically, CLIP studies and motif analysis provide evidence that Elav and its characteristic U-rich binding sites are enriched downstream of proximal PAS that are prone to bypass in neurons or in settings of ectopic ELAV/Hu RBPs (Carrasco et al., 2020; Wei et al., 2020) (Figure 3C). Prior *in vitro* tethering assays indicated that Elav can promote PAS bypass when recruited downstream of a cleavage site (Hilgers et al., 2012), and current studies extend that induction of 3' UTR lengthening by ELAV/Hu RBPs occurs in chromatin-associated nascent RNAs (Wei et al., 2020; Lee et al., 2021). Finally, striking genetic evidence for the importance of splicing regulation of *fne* by Elav in controlling neural APA came with the study of a specific deletion of the *fne* microexon by the Hilgers lab. This allele abrogated nuclear Fne accumulation in *elav* mutant embryos, and it enhanced the regulatory defects of *elav* mutants despite the overall normal levels of cytoplasmic Fne (Carrasco et al., 2020). These studies solidify the regulatory interplay between *Drosophila* ELAV/Hu family members in regulating the alternative neural transcriptome. Moreover, they are particularly striking given that neural APA is one of the first tissue-specific APA landscapes known to be directed by tissue-specific trans-acting factors, one of which (Fne) has only subtle defects on its own but turns out to be a critical regulator when its paralog Elav is not available.

## Open Mechanistic Questions for How ELAV/Hu RNA Binding Proteins Direct Neural Transcriptomes

### Molecular Bases for How ELAV/Hu RNA Binding Proteins Regulate the Alternative Neural Transcriptome

Extensive transcriptomic analyses coupled with molecular/cell biology and *Drosophila* genetics have revealed that ELAV/Hu family members are necessary and sufficient to direct neural splicing and APA patterns (Carrasco et al., 2020; Lee et al., 2021). For ALE splicing and tandem APA, this seems to be explained at least in part through local suppression of proximal PAS (Lisbin et al., 2001; Soller and White, 2003; Hilgers et al., 2012). However, we still lack clear molecular bases for how ELAV/Hu family RBPs recognize and mediate specific PAS choices, or how they mediate alternative cassette exon choices (both inclusion and exclusion). Mammalian HuR was proposed to inhibit association of the U2AF65 with the 3' splice site to mediate alternative splicing of *Fas* (Izquierdo, 2008), and HuR was also proposed to mediate PAS bypass by interfering with CstF-64 recruitment (Zhu et al., 2007; Dai et al., 2012). However, these have not been shown as general mechanisms. Without further direct mechanistic evidence, we cannot rule out that either other factors might be involved, or that perhaps some of these alternative splicing and 3' UTR changes are induced as secondary regulatory programs downstream of ELAV/Hu RBPs. In addition, while splicing and CPA are co-transcriptional processes, it is not ruled out that alternative splicing and APA programs might involve post-transcriptional regulation. For instance, differential stability of different isoforms, particularly of 3' UTR isoforms, could change the APA landscape. In fact, it was reported that mammalian HuD selectively stabilizes the long 3' UTR isoform of *BDNF* in neurons, via target sites in the distal *BDNF* 3' UTR (Allen et al., 2013). Thus, biochemical data that could better link ELAV/Hu factors to alterations in the activities of CPA or splicing machinery will be desirable (Figure 3). These could be achieved, for example, by combining proteomic analysis of ELAV/Hu factors, detailed structure-function studies using model target reporters, and dynamic profiling of nascent transcripts under conditions of ELAV/Hu manipulation.

At the heart of ELAV/Hu RBP function is the notion that they are recruited to relevant targets via their sequence-specific binding activity. While specific PAR-CLIP maps of mammalian HuR have been available for some time (Lebedeva et al., 2011; Mukherjee et al., 2011),there is currently far more limited resolution and depth of neural-restricted ELAV/Hu RBPs, including for *Drosophila* Elav and Fne CLIP (Alizzi et al., 2020; Carrasco et al., 2020; Lee et al., 2021) and mammalian nELAVL factors (Ince-Dunn et al., 2012; Scheckel et al., 2016). All of these studies generally suggest that metazoan ELAV/Hu RBPs identify similar U-rich binding sites, but till now it is difficult to use these maps to predict regulated targets and they have mostly been used in correlative manners. One key concern is that these procedures may preferentially capture more stable interactions on mature cytoplasmic mRNAs, and it is known that ELAV/Hu RBPs are shuttling factors. Therefore, it may be important to conduct such experiments so as to preserve information on nuclear vs. cytoplasmic association. Subcellular transcriptome mapping of the binding sites of individual ELAV/Hu RBPs may help to identify fundamental *cis*-elements associated with specific groups of PASs which are bypassed under the regulation of Hu family proteins, and whether there are distinctions between internal alternative splicing targets for exclusion or inclusion.

### Do ELAV/Hu RNA Binding Proteins Regulate Neural Alternative Polyadenylation Locally at Poly(A) Signals, or via Distant Genomic Association?

Elav has been proposed to regulate global neural 3' UTR extensions by a co-transcriptional recruitment strategy, in particular by associating with paused Pol II at GAGA-bearing promoters (Fuda et al., 2015; Oktaba et al., 2015). Evidence for this includes the fact that a minigene reporter bearing the native *elav* promoter can support Elav-dependent 3' UTR lengthening in an ectopic context, but that a synthetic promoter did not. This suggested that only certain promoters are amenable to ELAV responsiveness, and motif enrichment analysis pointed to GAGA motifs as a potential determinant. Second, Elav ChIP-seq analysis suggested that Elav localizes to paused promoters of neural TUTR-APA targets, and potentially to specific promoters of some genes with alternative transcription starts and 5' exon isoforms (Oktaba et al., 2015). This suggested a model that Elav is recruited to paused promoters, which pre-determines those pre-mRNAs as being responsive to bypass of non-neural PAS (Hilgers et al., 2012; Oktaba et al., 2015).

While an appealing model that Elav is pre-loaded onto the elongation complex, and thus prepared to inhibit CPA machinery, there are some potential concerns. First, the non-cognate promoter tested was the *Drosophila* Synthetic Core Promoter (DSCP) (Pfeiffer et al., 2008), and it remains to be seen other reporters linked to native promoters are similarly non-responsive to Elav. Second, although neural 3' UTR extension was attributed to association Elav at specific alternative promoters, the short-read sequencing technology used could not link such inferred transcript models directly. This remains to be validated using long-read or direct RNA sequencing. Third, another concern regards the specificity of Elav ChIP signals at promoters. For example, well-expressed genes can yield “hyper-ChIPable” artifacts at promoters (Teytelman et al., 2013).

In the future, additional experiments will be helpful to determine the contribution of paused promoter-mediated recruitment of Elav to its functional targets. In particular, it may be informative to assess Elav-mediated 3' UTR extension under conditions of genetically reduced pausing (e.g. by manipulating GAGA or the pause-inducing NELF complex). Moreover, now that Fne is also recognized as a critical factor in neural TUTR-APA, the recruitment mechanism may be expected to accommodate Fne as well. Finally, the model should accommodate if promoter recruitment of ELAV/Hu members can distinguish amongst multiple functional outcomes, including alternative cassette exon splicing, ALE splicing and tandem 3' UTR isoforms.

### Potential Role of Mammalian ELAV/Hu RNA Binding Proteins in Directing Neural Alternative Polyadenylation

Amongst numerous setting-specific APA programs that have been documented in mammals, we know of relatively few trans-acting factors (i.e., other than CPA machinery itself) that directly alter 3' UTR landscapes (Jenal et al., 2012; Batra et al., 2014; Bao et al., 2016; Yang et al., 2020). The highly extended 3' UTR landscape of the nervous system is one of the most profound tissue-specific APA profiles known and is conserved between flies and mammals (Smibert et al., 2012; Miura et al., 2013), yet its endogenous regulation by ELAV/Hu RBPs on the transcriptome scale was recognized recently (Hilgers et al., 2012; Carrasco et al., 2020; Wei et al., 2020).

The approaches used to dissect *Drosophila* ELAV/Hu family RBPs inform the ongoing study of their mammalian counterparts. On the loss-of-function side, the fly genetics clearly indicate that ELAV/Hu RBPs can have overlapping activities in mRNA processing. Metazoan ELAV/Hu RBPs bind relatively similar sequences, and mammalian neural ELAV/Hu paralogs are sufficiently similar that available antibodies do not distinguish them well. Thus, while nearly all genetic studies of mammalian ELAV/Hu RBPs manipulate only a single member, it seems that it is critical to consider the possibility of overlapping activities. Consistent with this, HuC/D dKO mice exhibit phenotypic enhancement over the single mutants (Ince-Dunn et al., 2012), but the triple *HuB/C/D* mutant awaits study. Moreover, since HuR has also been implicated in APA regulation of individual genes (Zhu et al., 2007; Dai et al., 2012; Mansfield and Keene, 2012), we cannot rule out that this ubiquitous ELAV/Hu member might also modulate the neural alternative transcriptome. Perhaps CRISPR/Cas9 methods may facilitate the analysis of such multiple knockout conditions.

On the other hand, the fact that ectopic expression of *Drosophila* ELAV/Hu RBPs can confer neuronal-like splicing and APA profiles to non-neural cell types (Oktaba et al., 2015; Wei et al., 2020; Lee et al., 2021) indicates that gain-of-function approaches are a viable alternative to interrogate the activities of an overlapping family. Collections of mammalian RBP expression constructs are available, even in a tethered format (Luo et al., 2020), and could be used to test their capacity to induce lengthened 3' UTRs.

### Other Potential Factors and Mechanisms for Neural Alternative Polyadenylation?

In our analysis of dissected *elav/fne* double mutant larval CNS, still about 1/3 of TUTR-APA targets largely maintained their neural extended 3' UTRs. This may suggest that other factors are involved in generating the neural extended 3' UTR landscape in *Drosophila*.

One obvious candidate is the third ELAV/Hu member Rbp9, since our ectopic studies in cultured cells showed that it is broadly capable of inducing alternative splicing, distal ALE isoform switches, and extended 3' UTR isoforms that largely reflect neural isoforms (Wei et al., 2020; Lee et al., 2021). From developmental proteomics, Rbp9 remains low during embryogenesis and larval stages, but increase during pupal and adult stages (Kim et al., 2010; Wei et al., 2020). Thus, even though *elav/fne* double mutants abolish most neural extended 3' UTRs in embryos and early larvae (Carrasco et al., 2020; Wei et al., 2020), Rbp9 might play roles in later stages. Additional combinatorial mutant analysis of the three ELAV/Hu members is warranted, especially during pupal or adult stages where Rbp9 levels are much higher. This would require complex genetics to bypass the early larval lethality of single *elav* mutants, but it could be possible using multiplex somatic CRISPR genetics.

It is also possible that other classes of trans-acting factors are involved (Figure 3). Existing studies of ELAV/Hu RBPs set relevant precedents, in that Elav/Fne/Rbp9 are all sufficient to induce neural isoform landscapes (Wei et al., 2020; Lee et al., 2021). Therefore, further gain-of-function screening for other neural-enriched factors that could induce neural APA could reveal new players in this process. Not only could this be monitored by the expression of individual targets or reporters, the availability of a collection of tethered mammalian RBP constructs (Luo et al., 2020) could serve as a platform for systematic screening on different types of model reporters. Alternatively, knowing that *fne* mutants strongly enhance *elav* mutants, one might consider loss-of-function approaches to reduce other neural regulators in the background of *elav* mutation. Again, given that combinatorial genetics can be time-consuming, having access to F1 screening involving efficient conditional Elav ablation (e.g., using RNAi or somatic CRISPR) could greatly facilitate such an endeavor.

Finally, it is worth considering the potential involvement of core CPA factors on tissue-specific APA. Since mRNA 3' ends involve differential recognition and utilization of multiple PASs in an individual transcription unit, it is reasonable to infer that the modulation of CPA factors might influence APA. For example, knockdown studies of a couple dozen individual CPA and splicing factors (Kubo et al., 2006; Martin et al., 2012; Yao et al., 2012; Li et al., 2015; Schwich et al., 2021) collectively reveal many conditions that exhibit global shifts towards proximal or to distal PASs. Thus, the balance of individual CPA factors, presumably affecting efficiency of CPA reactions, can plausibly regulate APA. Although many of these aforementioned tests involve deliberate depletion of CPA factors, there are increasing reports that endogenous modulation of different CPA factors is causal to different disease and cancer, including of CFIm25 (Masamha et al., 2014; Alcott et al., 2020), Pcf11 (Ogorodnikov et al., 2018) and the CPSF complex (Chen et al., 2021).

### Physiological and Pathological Significance of ELAV/Hu-Regulated Neural Alternative Polyadenylation

Beyond the mechanics and genomics of generating tissue-specific alternative transcriptomes, a fundamental question remains about whether this matters for normal biology? It is clear that alternative splicing creates new isoforms with critical functions, but it is somewhat less clear how broad the phenotypic impacts of 3' UTR isoforms are, especially within endogenous contexts. Since 1) hundreds of genes are processed into longer 3' UTR isoforms specifically in neurons, 2) these are often kilobases in length, 3) these frequently utilize conserved, canonical PAS, 4) the overall feature of extended neural 3' UTRs is broadly conserved between flies and mammals, it seems certain that neural APA is not a fortuitous process, but rather is implemented for specific regulatory and/or functional outcomes that are of utility in neurons. However, this does not directly implicate that alternative neural 3' UTRs are necessarily of phenotypically relevant developmental or behavioral consequences.

3' UTRs certainly do harbor substantial sites for miRNAs and RBPs, which can be demonstrated functional in a variety of experimental assays. However, there are increasing efforts that show that specific mutation of endogenous 3' UTR binding sites or of entire 3' UTRs did not recapitulate the expected effects (Bassett et al., 2014; Mitschka and Mayr, 2021). Thus, specific mutational experiments are critical to assess phenotypes. Now that we know that ELAV/Hu factors are global regulators of neural APA and splicing, we may expect phenotypic studies of these mutants (Simionato et al., 2007; Rogulja-Ortmann et al., 2014; Castro Alvarez et al., 2021), or combinations thereof (Ince-Dunn et al., 2012; Wei et al., 2020; Lee et al., 2021), to lend insight. On the other hand, the extremely broad programs of deregulated splicing and APA isoforms in such mutants also means that it will be challenging to assign specific molecular bases to such mutants, to say nothing of distinguishing direct from indirect effects. Thus, specific genetic alteration of endogenous 3' UTRs would be highly desirable.

**FIGURE 4:**
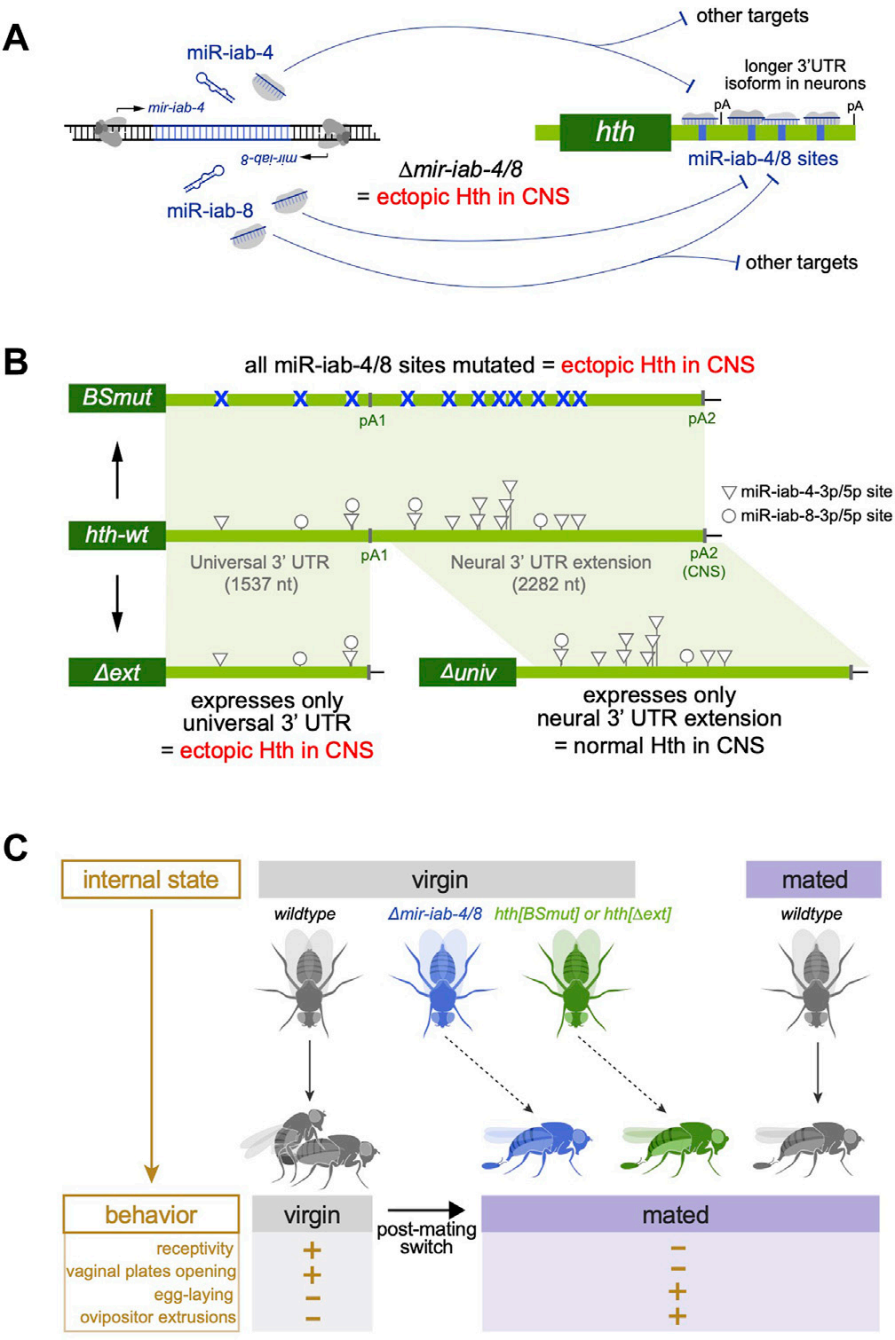
Biological impact of neural polyadenylation on development and behavior. **(A)** An individual hairpin locus in the *Drosophila* Bithorax Complex is bidirectionally transcribed to generate distinct miRNAs (miR-iab-4 and miR-iab-8) that are expressed in specific domains of the central nervous system (CNS). One of the major targets of these miRNAs in *homothorax* (*hth*), which contains numerous conserved binding sites for these miRNAs. *hth* is broadly expressed but undergoes 3' UTR lengthening in the CNS, creating a scenario for enhanced miRNA targeting in this setting. Expression of the *hth* 3' UTR extension is lost in *elav/fne* mutants, and Hth protein is ectopically expressed in the CNS of miRNA deletion flies. **(B)** Targeted genetic manipulation of the endogenous *hth* 3' UTR creates alleles bearing point mutations in all of the miR-iab-4/8 sites, or that delete either the proximal (universal) *hth* 3' UTR or the extended 3' UTR expressed in the nervous system. Both the *hth[BSmut]* and *hth[∆ext]* alleles derepress Hth in the CNS, but the *hth[∆univ]* allele has normal Hth. **(C)** Behavioral assays demonstrate that dual regulation of *hth* by miR-iab-4/8 and neural alternative polyadenylation is required to match the behavior of virgin females with their internal state. In wildtype, virgin females are sexually receptive and tend not to lay eggs, but after mating, females oppose male courtship and increase egg laying. Virgin females of *∆mir-iab-4/8*, *hth[BSmut]*, and *hth[∆ext]* genotypes all exhibit mated-like behaviors.

The power of *Drosophila* genetics has been informative in this regard. For example, using transgene rescues, the extended neural 3' UTR of the calcium/calmodulin-dependent protein kinase II gene (*CaMKII*), which was shown to be required for normal synaptic plasticity (Kuklin et al., 2017). In a more complicated example, the extended neural 3' UTR of *Dscam1* is induced by Elav/Fne, and somehow coupled to skipping of an internal exon (Zhang et al., 2019). Notably, a specific deletion of the extended *Dscam1* 3' UTR selectively ablates the long but not short isoform, affects *Dscam1* alternative splicing, and causes axonal projection defects. Finally, we found that the distal 3' UTR of the transcription factor *homothorax* (*hth*) enables regulation by the neural miRNA locus *mir-iab-4/8*, since many high affinity sites are located within its neural-specific 3' UTR extension (Garaulet et al., 2014; Garaulet et al., 2020) (Figure 4A). Remarkably, a linear regulatory pathway emerges from the analysis of specific fly mutants that are deleted for *mir-iab-4/8*, are specifically mutated for miR-iab-4/8 binding sites in the *hth* 3' UTR, or are deleted for the *hth* neural 3' UTR extension (Figure 4B). Female mutants of all three genotypes derepress Hth protein in the abdominal ganglion of the ventral nerve cord and exhibit specific behavioral defects. In particular, virgin females of all three mutants behave as if they were subjectively mated (Garaulet et al., 2020), in part through downregulation of the transcription factor Doublesex (Garaulet et al., 2021). Thus, miRNA binding sites and neural APA act on a single gene to confer appropriate behavior (Figure 4C). Intriguingly, expression of the neural *hth* 3' UTR extension in the larval CNS depends on the combined activities of Elav/Fne (Wei et al., 2020), implying that ELAV/Hu RBPs mediate adult female behavior via *hth*.

Several exciting precedents for endogenous functions of neural 3' UTRs and/or explicitly neural APA also exist in mice. For example, the long neural 3' UTR of Importin β1 mediates its axonal localization, and specific deletion of this region impairs recovery from nerve injury (Perry et al., 2012). In another recent example, a deletion allele of the neural 3' UTR extension of the Ca^++^ regulator *Calmodulin* 1 (*Calm1*) was generated. This mouse mutant maintains expression of the proximal 3' UTR isoform, but exhibits disorganized dorsal root ganglion and reduces *Calm1* expression (Bae et al., 2020).

There is no shortage of loci to nominate for hypothesis-driven assessments, e.g., to remove endogenous 3' UTRs or specifically of alternative neural 3' UTR extensions. With the obvious suitability of CRISPR/Cas9 for this task (Zhao et al., 2017; Terenzio et al., 2018; Thomas et al., 2020), we may look forward more animal studies of alternative 3' UTR deletions. Key to this may be higher throughput isoform-specific deletion strategies, perhaps in the context of *in vitro* differentiated neurons (Bae and Miura, 2021). These may enable screening for alternative isoforms with overt phenotypic impacts, which could complement efforts to generate fly and mouse 3' UTR mutants. With already these initial studies showing that individual extended neural 3' UTRs are required for different aspects of neural specification, differentiation, and/or behavior, we may expect the coming years to expand the phenotypic requirements of these alternative isoforms during normal contexts and in neurological disease and cognitive decline. Reciprocally, given that at least some ELAV/Hu RBPs are sufficient to induce neural splicing and 3' UTR isoforms, it is also conceivable that their aberrant or ectopic expression could also underlie pathological conditions.

## Conclusion

Neural ELAV/Hu genes and their RBP products have been studied since the mid 1980s, starting with *Drosophila* genetics and extending to human autoantigens. Since then, we have learned a great deal on their biological requirements in diverse aspects of neural specification, differentiation and behavior. In addition, we also have learned that ELAV/Hu factors broadly determine multiple aspects of neural expression and the alternative neural transcriptome, including usage of alternative cassette exons, distal alternative exons, and extended 3' UTR isoforms. Further knowledge on the biochemical and genetic functions of ELAV/Hu RBPs will illuminate basic mechanisms of mRNA processing, development and function of the nervous system, and likely human neurological disorders.
